# Necrology

**Published:** 1885-03

**Authors:** 


### Necrology.

Died, at the house of her uncle, Dr. C. Bronson, in Cincinnati, on Wednesday morning, February 11th, 1885, of typho malarial fever, Miss Donna M. Shadoan, the youngest daughter of the late Dr. W. H. Shadoan, of Kentucky.

The demise of the young is always an occasion for sorrow, but in some cases more than in others; because of loveliness of person and character, Miss S. was the favorite of all who knew her, and was beloved by every one. She reflected sunshine and happiness upon all with whom she came in contact. Her habit was to perceive and enjoy the pleasant side of almost every condition and circumstance of life, and to see beauty where others see only the opposite. The members of the family, who are thus so sorely bereaved, have the heartfelt sympathy of not only their friends, but of hers as well.

